# How does parental involvement influence students’ wellbeing? A moderated mediated model

**DOI:** 10.3389/fpsyg.2026.1733507

**Published:** 2026-01-22

**Authors:** Qiu Yan Zhang, Nurwina Anuar

**Affiliations:** 1 Faculty of Education, Universiti Kebangsaan Malaysia, Bangi, Malaysia; 2 Faculty of Education and Livelihood, Xiamen City University, Xiamen, China

**Keywords:** gender difference, home-school collaboration, moderated mediation, parental involvement, psychological wellbeing, subjective wellbeing

## Abstract

Parental involvement has been widely associated with adolescents’ wellbeing, yet the psychological mechanisms underlying this association remain incompletely understood. Drawing on self-determination and family-school system perspectives, the present study examined the mediating role of psychological wellbeing and the moderating role of gender in the association between parental involvement and subjective wellbeing among Chinese adolescents. Participants were 450 junior middle school students recruited through stratified cluster random sampling. Structural equation modeling and conditional indirect effect analyses were used to test a moderated mediation framework. The results showed that parental involvement was positively associated with both psychological wellbeing and subjective wellbeing. Psychological wellbeing partially mediated the association between overall parental involvement and subjective wellbeing and fully mediated the effect of adolescents’ attitudes toward parental involvement. Gender significantly moderated the strength of the indirect effect in the overall model, particularly for behavior-oriented forms of parental involvement, with stronger indirect associations observed among girls. In addition, several demographic characteristics, including grade level, parental marital status, co-residence status, and parental education, were associated with levels of parental involvement and wellbeing. These findings underscore the importance of adolescents’ subjective interpretations of parental involvement and highlight gender as a boundary condition in the psychological processes linking family involvement to wellbeing. Although the findings are correlational, they contribute to a more differentiated understanding of parental involvement and adolescent wellbeing from a developmental perspective.

## 1 Introduction

Over decades of development in positive psychology, wellbeing has increasingly become a key indicator of a society’s prosperity and vitality, as well as a crucial measure of an individual’s health status (Biswas-Diener et al., 2004; Chue and Yeo, 2023). Students in junior middle school are at a critical stage in life, where wellbeing levels lay the foundation for healthy psychological development in adulthood (Kerestes and Stulhofer, 2020). However, the student’s mental health and wellbeing during this period are not encouraging (World Health Organization, 2022). Various sectors of society are seeking ways to enhance students’ wellbeing (Alsarrani et al., 2022; Arslan, 2024). As the two most significant environmental factors in adolescent development, family and school exert a pivotal influence on mental health and wellbeing (Tudge et al., 2016). Establishing strong home-school partnerships has also become a key strategy for the education sector worldwide to enhance student wellbeing (Organisation for Economic Co-operation and Development [OECD], 2024).

Parental involvement refers to the cooperation between families and schools to support student learning, development, and long-term success (Epstein and Boone, 2022; Epstein, 2010; Hill, 2022). Family and school are two central ecological contexts for adolescents, and their influences on mental health can reinforce each other or, in some cases, come into conflict (Tudge et al., 2016). To promote coordinated efforts across these systems, various national and regional policies have emphasized the importance of home–school partnerships (Australian Government, 2013; National People’s Congress Standing Committee, 2021; United Kingdom Department for Children Schools Families, 2009; United States Congress, 2002). Evidence suggests that parental involvement contributes to adolescents’ academic, emotional, and behavioral adjustment (Australian Government, 2013; Hill, 2022). However, as adolescents gain autonomy, they may resist certain forms of parental involvement, making effective family–school collaboration more complex at this developmental stage (Epstein, 2010; Salinas et al., 2019). Despite growing interest in parental involvement, the mechanisms through which it influences student wellbeing remain insufficiently defined, and research examining potential moderators of this relationship (e.g., gender) remains limited (Aldridge and McChesney, 2018; Okagbue et al., 2025; Yang et al., 2023).

Similarly, the Chinese government has introduced a series of policy measures to strengthen family-school partnerships, recognizing their crucial role in education (Ministry of Education of the People’s Republic of China, 2023; National People’s Congress Standing Committee, 2021). However, the existing research in China has primarily focused on theoretical discussions at the experiential level, lacking studies on parental involvement among junior middle school students (Yang et al., 2023). At the same time, as a developing country in Asia, China still has gaps in research on the wellbeing of adolescents (Aldridge and McChesney, 2018). Furthermore, empirical research on the causal pathways linking parental involvement to student wellbeing remains insufficient (Li et al., 2020; Liu, 2023).

Therefore, this study focuses on adolescent students to explore the relationship between parental involvement and student wellbeing. Building upon self-determination theory, it further examines the mediating and moderating effects of basic psychological needs fulfillment and demographic variables. The specific research hypothesis are as follows:

*H1*: The levels of students’ subjective wellbeing and psychological wellbeing are significantly different from the population average.

*H2*: The level of parental involvement is significantly different from the population average.

*H3*: Psychological wellbeing partially mediates the relationship between parental involvement and subjective wellbeing.

*H4*: Gender moderates the indirect relationship between parental involvement and subjective wellbeing via psychological wellbeing, with the effect being stronger for female students.

### 1.1 Literature review

Wellbeing encompasses both momentary pleasure and the optimization of psychological functioning, as well as the quality of experience (Alsarrani et al., 2022). Wellbeing has long been seen as a key factor in building and sustaining a healthy and productive society (Chue and Yeo, 2023; Diener and Suh, 1997; Kerestes and Stulhofer, 2020). With a shared understanding of the importance of wellbeing, people’s definition of health has also undergone a significant shift. In 2020, the World Health Organization redefined health through the lens of positive psychology. It states that health is a state of wellbeing characterized by the capacity to express, think, interact, earn a living, and enjoy life. In this state, individuals recognize their ability to work effectively, cope with normal life stresses, and contribute to their communities (World Health Organization, 2022).

Two main indicators are often used to measure wellbeing: Subjective wellbeing and psychological wellbeing (Garcia et al., 2017; Goodman et al., 2022; Newland et al., 2019; Ryan and Deci, 2001). Subjective wellbeing refers to how individuals evaluate their own lives (Alsarrani et al., 2022; Biswas-Diener et al., 2004). It typically refers to a state of happiness, encompassing positive emotions, fewer negative emotions, and higher life satisfaction. It reflects the cognitive and affective components of happiness (Alsarrani et al., 2022). It can also be seen from this definition that the affective component (positive and negative emotions) is linked to real-life stressors and exhibits greater fluctuation, while the cognitive component (life satisfaction) tends to be more stable (Amholt et al., 2020; Martela and Sheldon, 2019). This also reflects the fluctuating and stable characteristics of subjective wellbeing.

Psychological wellbeing refers to the capacity to pursue happiness, encompassing the realization of personal potential through multidimensional variables such as self-actualization, meaning, and vitality (Gandarillas et al., 2024; Grantham-McGregor et al., 2014; Stavraki et al., 2022). It focuses on personal growth and realizing one’s full potential, also on the ability to sustain positive emotional states (Stavraki et al., 2022). psychological wellbeing is recognized as a primary factor in optimal human functioning (Ryff and Keyes, 1995). It is occasionally investigated as a predictor or prerequisite for individual positive development, but also as an outcome of high levels of subjective wellbeing (Amholt et al., 2020; Stavraki et al., 2022). A growing number of researchers acknowledge that these two indicators represent distinct, non-substitutable dimensions of wellbeing (Martela and Sheldon, 2019; Gandarillas et al., 2024).

Studies across different countries have consistently found that during early to mid-adolescence (ages 10–16), there is a significant decline in the cognitive dimensions of positive emotions and wellbeing (such as overall life satisfaction), while negative emotions tend to increase (Cassidy and Boulos, 2023; El-Abbassy et al., 2020). Globally, around 23–24% of young people aged 10–19 face psychological or behavioral problems. Half of these issues begin before the age of 14 (World Health Organization, 2022). The detection rate of depression risk among the junior middle school students was 26.6% in China (Cheong et al., 2023). As crucial developmental environments for adolescent students, families and schools harbor numerous factors influencing student wellbeing. These include parenting styles, parent-child communication patterns, family atmosphere, school climate, teacher-student relationships, and peer relationships (Francis et al., 2021; Gandarillas et al., 2024; Schoeps et al., 2023). Therefore, to better enhance adolescents’ mental health and wellbeing, the state and educators are actively promoting the widespread implementation of parental involvement in education (Ministry of Education of the People’s Republic of China, 2023).

Parental involvement, which is called family-school cooperation in the Chinese context, refers to the synergistic educational efforts achieved through mutual coordination, concerted cooperation, and shared direction between families and schools (Wang et al., 2022). It is also known as the family-school partnership or parental engagement. It primarily refers to the collaborative relationship between parents and schools centered on student academic achievement and development to achieve educational goals and future success (Epstein and Sanders, 2006; Hill et al., 2018). Existing research indicates that home-school collaboration enhances children’s educational achievement (Gandarillas et al., 2024; Hill, 2022; Yang et al., 2023) and promotes their social-emotional development (Abín et al., 2024; Wild, 2018), reducing behavioral and mental health issues (Aleid et al., 2024; Okagbue et al., 2025), and enhancing wellbeing (Liu et al., 2024; Thomas et al., 2020).

Parental involvement can be situated within the family–school relationship model proposed by Adams et al. (2017); Ali et al. (2022); Ryan and Adams (1995), which is grounded in an ecological systems perspective. This framework places child outcomes at the center and conceptualizes family and school influences as operating across proximal and distal levels. It posits that family processes influence educational and developmental outcomes primarily through the child’s own psychological characteristics and interpretations. Accordingly, proximal experiences—such as parent–child interactions and daily forms of support—are expected to exert stronger and more immediate effects than distal contextual factors (Ali et al., 2022).

Within this framework, parental involvement is best understood as a multidimensional construct embedded in the family–school system, whose effects are shaped not only by parental behaviors themselves but also by how these behaviors are perceived and internalized by adolescents. Rather than assuming that parental involvement uniformly translates into developmental benefits, the model highlights the mediating role of students’ subjective perceptions in linking parenting practices to psychological outcomes. These perceptions influence whether parental involvement is experienced as supportive, autonomy-enhancing, or controlling, thereby shaping adolescents’ psychological functioning and wellbeing.

At the same time, Self-Determination Theory (SDT) provides a clear mechanism for understanding how parental involvement influences students’ psychological and subjective well-being (Adams et al., 2017). It proposes that external environments shape well-being primarily through the satisfaction of three basic psychological needs: autonomy, competence, and relatedness. When these needs are fulfilled, individuals experience higher levels of vitality and psychological functioning, which subsequently promote broader forms of wellbeing (Ryan and Deci, 2001). Parental involvement, particularly when enacted in a warm, structured, and autonomy-supportive manner, represents a salient social context in adolescents’ daily lives and thus constitutes a key source of need support. Such involvement can enhance adolescents’ sense of volitional agency (autonomy), perceived effectiveness in managing academic and life demands (competence), and feelings of emotional connection and belonging within the family (relatedness) (Ryan and Deci, 2019).

Importantly, the experiential outcomes are closely reflected in the core dimensions of Ryff’s psychological wellbeing framework, including autonomy, environmental mastery, personal growth, positive relations with others, purpose in life, and self-acceptance (Chan et al., 2019; Ryan and Deci, 2019; Stavraki et al., 2022). Although SDT needs satisfaction and psychological well-being are conceptually distinct, the two frameworks converge at the level of psychological functioning. From this perspective, PWB can be conceptualized as a proximal psychological pathway through which parental involvement exerts its influence on broader evaluations of life quality. By linking broader behavioral and environmental factors to personal experiences, they explain a significant portion of the variance in well-being (Martela and Sheldon, 2019; Ryan and Deci, 2019). This integrative pathway is consistent with empirical evidence demonstrating that need-supportive parenting predicts both psychological functioning and subjective well-being outcomes across developmental contexts (Ryan and Deci, 2019; Stavraki et al., 2022).

Accordingly, prior research can be broadly classified into studies focusing on parental involvement as a global construct, studies emphasizing behavioral participation, and a smaller body of work examining students’ attitudes toward parental involvement. Most existing studies rely on parent- or teacher-reported measures of parental involvement (Rogers et al., 2009; Thomas et al., 2020). Such reports may not fully capture students’ actual experiences of parental engagement. Students’ perceptions of parental involvement, including their attitudes toward parental support and their recognition of specific parental behaviors, often differ from parents’ self-reports (Thomas et al., 2020). It includes students’ attitudes toward parental involvement and their understanding of parental involvement-related behaviors. Parental involvement-related behaviors can be divided into home-based involvement (e.g., homework help, monitoring, and school-related conversations) and school-based involvement (e.g., attending school events or communicating with teachers) (Hill, 2022). Therefore, measuring parental involvement from the student perspective offers a more proximal and experience-based understanding of how parental involvement relates to student wellbeing.

Research also suggests that the effect of parental involvement may vary by gender, making gender a theoretically meaningful moderator. Prior studies have shown that boys and girls differ in socialization patterns, emotional expression, and their sensitivity to parental support or control (Careemdeen, 2024; Wilson Fadiji and Reddy, 2023). Some studies report that girls benefit more from parental warmth and autonomy support, whereas boys respond more to structured guidance and performance-oriented involvement (Careemdeen, 2024). However, other studies find no gender differences (Wilson Fadiji and Reddy, 2023) or even opposite patterns (Chan et al., 2019), indicating that the moderating role of gender is far from consistent across contexts.

Additionally, from the perspective of Self-Determination Theory, boys and girls may experience and interpret parental behaviors differently, leading to variations in the extent to which autonomy, competence, and relatedness needs are met (Alijev et al., 2021). Because basic need satisfaction is central to the pathway linking parental involvement to psychological wellbeing, gender-based differences in need fulfillment may result in differential effects on psychological wellbeing and subsequently subjective wellbeing. Therefore, examining gender as a moderator is both theoretically grounded and necessary for clarifying mixed findings in the literature.

However, research on junior middle school students’ wellbeing (Aldridge and McChesney, 2018), parental involvement (Yang et al., 2023), and the relationship between the two (Liu et al., 2024) remains in its infancy in China, with theoretical gaps persisting. As an external environmental influence, the pathways through which parental involvement affects students’ subjective experiences of wellbeing remain unclear. To address these gaps, the present study investigates how various forms of parental involvement relate to junior middle school students’ wellbeing. Drawing on Self-Determination Theory, we further examine whether the fulfillment of basic psychological needs, captured here as psychological wellbeing, mediates the relationship between parental involvement and subjective wellbeing. In addition, given potential gender-based differences in adolescents’ perceptions of parental behaviors, the study tests gender as a moderator in this pathway.

## 2 Materials and methods

### 2.1 Participants

A stratified cluster random sampling method was employed to recruit participants. The target population consisted of junior middle school students in Xiamen across three school districts. Within each district, two schools were selected through convenience sampling based on accessibility and the school’s willingness to participate. At each school, 80 students were randomly selected from the official student roster using sequential numbers. The selection included 30 students each from Grades 7 and 8, and 20 students from Grade 9.

With approval from school administrators and parental consent, 480 questionnaires were distributed, and 467 were returned. Before analysis, questionnaires were screened for completeness and response validity. Cases were excluded if: (a) more than 20% of items on any major scale (parental involvement, psychological wellbeing, or subjective wellbeing) were missing, (b) the response pattern indicated inattentive responding (e.g., identical answers for long sections), or (c) demographic information essential for subgroup analysis was missing. After applying these criteria, 450 valid questionnaires remained, yielding a valid response rate of 96.4%. For cases with minor item-level missing data ( ≤ 20% within a scale), mean imputation using the participant’s available items on the same scale was applied. No variable exceeded 5% missing data after screening. Sensitivity analyses using multiple imputation procedures produced results consistent with the primary analysis.

The final sample consisted of 50.7% male and 49.3% female students. Grade distribution was 40.7% in Grade 7, 40.4% in Grade 8, and 18.9% in Grade 9. Regarding birth order, 20.2% were only children, 44.2% were firstborns, 32.6% were second children, and 2.9% had three or more siblings. Most students (90.7%) had married parents; 9.3% reported parental separation, divorce, or widowhood. Concerning living arrangements, 79.1% lived with both parents, 14.0% with their mother only, 4.3% with their father only, and 2.7% with other relatives. Parental education levels varied, with 37.6% of fathers and 41.6% of mothers holding a high school education or below, 37.4% of fathers and 30.9% of mothers holding a college degree, and 7.3% of fathers and 6.4% of mothers holding a master’s degree or above.

### 2.2 Instruments

#### 2.2.1 Subjective wellbeing

Subjective wellbeing was accessed by World Health Organization Wellbeing Index (WHO-5). This brief self-report questionnaire assesses subjective wellbeing over the past 2 weeks. It comprises five self-rated items related to different emotional states. These five positively worded items are scored on a Likert scale ranging from 0 (never) to 5 (always). The total raw score ranges from 0 to 25, which is then multiplied by 4 to convert to a percentage (0–100). Higher scores indicate greater wellbeing. A score of 50 or below indicates poor wellbeing and is commonly used for depression screening. In Chinese populations, Cronbach’s alpha coefficients (Chan et al., 2021) for both the total score and individual items range from 0.91 to 0.93, indicating high reliability.

In this study, confirmatory factor analysis (CFA) was conducted using maximum likelihood estimation in AMOS 29. The model indicated that the single-factor model fit the data well: χ^2^(4) = 18.97, *p* = 0.000, CFI = 0.98, TLI = 0.96, RMSEA = 0.09. All standardized factor loadings ranged from 0.67 to 0.91 (*p* < 0.001), demonstrating that the measurement model possesses good validity. Internal consistency reliability in the current sample was satisfactory, with Cronbach’s α = 0.89.

#### 2.2.2 Psychological wellbeing

Psychological wellbeing was accessed by Brief Chinese version of Ryff’s Psychological Wellbeing Scale. This abbreviated version of the psychological wellbeing is used to assess adolescent mental health (Chan et al., 2019). It comprises six subscales: Autonomy, Environmental Mastery, Personal Growth, Relatedness, Life Purpose, and Self-Acceptance. The 24-item scale employs a Likert-type rating scale ranging from 1 (least like me) to 5 (most like me). The measured dimensions significantly explain overall variability in quality of life by revealing levels of mental health. Adolescents with higher scores are considered happier and more functionally well-adjusted. All six subscales of the 24-item Chinese version demonstrated good internal consistency, with Cronbach’s alpha coefficients ranging from 0.72 to 0.85.

In the present study, CFA was conducted using maximum likelihood estimation to verify the six-factor structure. The model demonstrated acceptable fit: χ^2^(237) = 791.93, *p* < 0.001, CFI = 0.91, TLI = 0.89, and RMSEA = 0.07. All standardized factor loadings ranged from 0.60 to 0.89 (*p* < 0.001), indicating good convergent validity. Internal consistency reliability in the current sample was satisfactory, with Cronbach’s α values ranging from 0.62 to 0.87 across the six subscales. Although most subscales demonstrated acceptable internal consistency, the self-acceptance dimension of psychological wellbeing showed relatively modest reliability in the present sample (Cronbach’s α≈0.62), a pattern that has been reported in prior studies using Chinese samples (Chan et al., 2019; Cheng and Chan, 2005).

#### 2.2.3 Parental involvement

Parental involvement was accessed by the scale of *Students’ Perception of Parental Involvement* (Hicks, 2023), which was originally developed and validated as a three-factor structure. The scale includes 18 items divided into the following three subscales: students’ attitudes toward parental involvement (5 items), family activities (5 items), and school activities (8 items). Students rated each item on a 4-point Likert scale ranging from 1 (strongly disagree/never) to 4 (strongly agree/daily), with higher scores indicating higher perceived parental involvement.

CFA was performed using maximum likelihood estimation to verify the original three-factor model. Model fit indices indicated an adequate fit: χ^2^132) = 899.498, *p* = 0.000, CFI = 0.88, TLI = 0.87, RMSEA = 0.09. Although CFI and TLI were slightly below the conventional 0.90 threshold, the RMSEA indicated borderline acceptable fit. Similar patterns have been commonly observed when applying validated instruments across different cultural or linguistic contexts (Cheng and Chan, 2005). No items were removed, and all standardized factor loadings ranged from 0.52 to 0.89 (*p* < 0.001), supporting convergent validity.

Internal consistency reliability for each subscale in this study was satisfactory: attitudes toward parental involvement (α = 0.72), family activities (α = 0.79), and school activities (α = 0.86). The overall scale demonstrated acceptable reliability, supporting its use in the current sample.

### 2.3 Ethics statement

The studies involving humans were approved by the Special Committee of Scientific Research Ethic of Liaocheng University (No. HE2025090401). The studies were conducted in accordance with the local legislation and institutional requirements. The participants provided their and their parents’ written informed consent to participate in this study.

### 2.4 Data collection procedure

This study adopted group testing. After obtaining the informed consent from school leaders and the students themselves, the selected students were gathered in an independent classroom for the test. Each class was assigned 1–2 administrators. All administrators were research staff who had received training on test administration operations. Before the test, the administrators read out the instructions. In the instructions, the significance of this survey was explained. It was also emphasized that the survey was anonymous and there were no right or wrong answers to the questions. The students were required to answer independently based on their actual situations. The time for the students to complete all the questionnaires was approximately 10-15 min.

### 2.5 Analysis plan

Data were analyzed using SPSS 29.0 and Amos 29.0. The analysis followed a multi-level, complementary strategy. This approach aimed to comprehensively address the research questions.

The first step is Preliminary Analysis and Data Validation.

Means and standard deviations were calculated for all key variables. These variables included three dimensions of parental involvement, psychological wellbeing, subjective wellbeing, and demographic variables. A Spearman correlation matrix was generated. It was used to conduct an initial examination of inter-variable relationships. Then, a Harman’s single-factor test was employed for *post-hoc* assessment of common method bias.

Next, CFA was utilized to examine the factor structure of the key construct of parental involvement. CFA was estimated using maximum likelihood (ML) (Hicks, 2023; Kline, 2016). Two models were compared for this validation: the first-order factor model (with the three dimensions of parental involvement correlated with one another, referred to as the dimension-specific model) and the second-order factor model (with a higher-order “overall parental involvement” factor, referred to as the overall effect Model). Then, a chi-square difference test was conducted to identify the optimal model. Finally, this validated measurement model was applied to subsequent analyses of overall effects.

Prior to conducting analyses that treated each dimension of parental involvement as a concurrent predictor, variance inflation factors (VIFs) and tolerance values were calculated. This step ensured the stability of regression coefficients in the subsequent analyses.

The second step is the Hypothesis Testing, including Main, Mediating, and Moderating Effects. Hypothesis testing adopted two complementary model specifications. These specifications were implemented via distinct statistical approaches.

**FIGURE 1:**
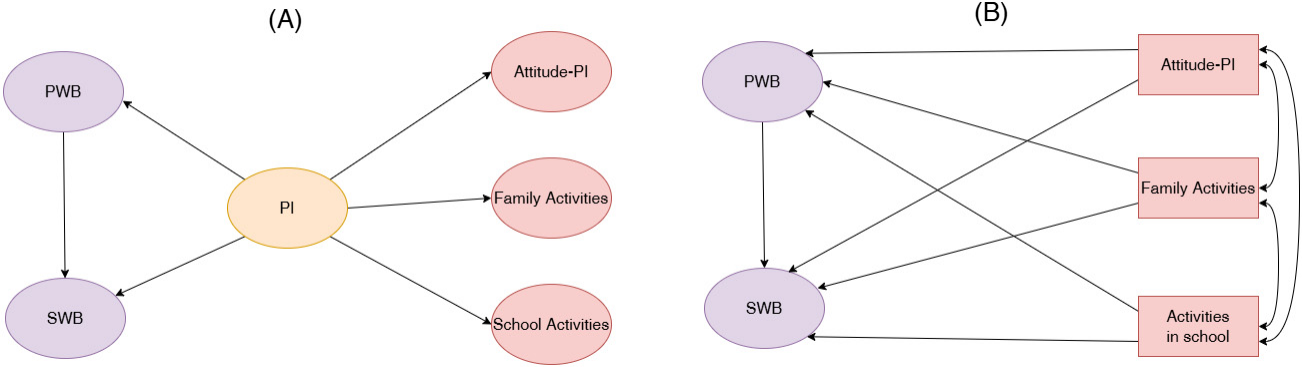
Conceptual models for hypothesis testing. **(A)** The overall latent variable model. **(B)** The dimension-specific path model. **(A)** Depicts the overall latent variable model, in which parental involvement (PI) is specified as a higher-order latent construct predicting psychological wellbeing (PWB) and subjective wellbeing (SWB), with PWB modeled as a mediator. **(B)** Illustrates the dimension-specific path model, in which attitudes toward parental involvement, activities at home, and activities at school are treated as correlated but distinct components of parental involvement and are simultaneously specified as predictors of PWB and SWB. Together, the two models represent complementary analytic frameworks for examining both overall and dimension-specific pathways linking parental involvement to adolescent wellbeing.

The Overall Effect Model was employed to test the impact of overall parental involvement on subjective wellbeing through psychological wellbeing. Meanwhile, the dimension-specific mediation analysis was conducted to examine whether each parental involvement dimension (Attitude toward parental involvement, Family activities, School activities) indirectly influences subjective wellbeing through psychological wellbeing. Path analysis will be performed within a structural equation modeling (SEM) framework using Amos 29. Three parallel mediation pathways will be specified—one for each parental involvement dimension—with psychological wellbeing as the mediator. Bootstrap confidence intervals (5,000 resamples) will be used to test the significance of the indirect effects. Model fit will be evaluated using standard indices. Standardized coefficients (β) will be reported for all direct and indirect paths. Figure 1 schematically illustrates the two core model specifications used for testing the hypotheses.

To examine whether gender moderates the aforementioned mediating paths, the moderation effect test was conducted. For the Overall Effect Model, multi-group structural equation modeling was used (Byrne, 2016; Kline, 2016). The approach compared whether path coefficients were equivalent across different gender groups. For the Dimension-Specific Model, the SPSS PROCESS macro was employed. Interaction terms were constructed within a regression framework. The moderation effects were then tested. Conditional indirect effect values across different groups were calculated and reported. The index of moderated mediation was also computed and presented. This allowed for a precise description of the specific stage at which the moderation effect occurred.

All analyses controlled for grade level and parental education, which are known correlates of adolescent wellbeing. Standardized estimates were reported for all SEM results.

## 3 Results

### 3.1 Control and testing of common method bias

Because all variables in this study were collected through self-report questionnaires, common method bias (CMB) was examined using two complementary approaches recommended by Podsakoff et al. (2003) and Podsakoff et al. (2013). Procedurally, we ensured respondent anonymity and varied response scales.

Statistically, Harman’s single-factor test was conducted by performing unrotated exploratory factor analysis (EFA) on all measurement items. Results showed that there were 5 factors with eigenvalues > 1, and the first factor accounted for 30.086%, which was below the critical threshold of 40%. Although this approach is limited and cannot conclusively rule out CMB, it provides initial evidence that no single factor dominated the variance.

Furthermore, the confirmatory factor analysis (CFA) comparison method was applied. A single-factor model, where all items loaded on one latent factor, showed poor fit (CFI = 0.589, TLI = 0.546, RMSEA = 0.137). In contrast, the hypothesized multi-factor measurement model demonstrated substantially better fit (CFI = 0.954, TLI = 0.944, RMSEA = 0.070). The significant deterioration in model fit supported the absence of substantial common method variance. Therefore, results from both the exploratory and confirmatory approaches indicated that common method bias was not a serious threat in this study.

### 3.2 Preliminary analyses

#### 3.2.1 Effects of gender and parental marital status

Independent samples *t*-tests (see Table 1) revealed significant differences between participants of different genders and parental marital status in parental involvement, subjective wellbeing, and psychological wellbeing.

**TABLE 1:** Independent samples *t*-tests for the effects of gender and parental marital status on wellbeing and PI.

Variables	Dimensions	M±SD	*t*	df	*p*	Cohen’s d
**Gender**						
SWB	Male	65.13 ± 25.41	5.081	448	< 0.001	0.48 [0.29, 0.67]
	Female	53.15 ± 24.61				
PWB	Male	3.62 ± 0.86	2.751	448	0.003	0.26 [0.08, 0.44]
	Female	3.41 ± 0.76				
PI	Male	2.14 ± 0.56	3.287	448	< 0.001	0.31 [0.12, 0.50]
	Female	1.98 ± 0.49				
**Parental marital status**						
SWB	Married	59.70 ± 29.70	1.208	448	0.114	0.18 [–0.04, 0.41]
	Apart	54.70 ± 26.00				
PWB	Married	3.54 ± 0.82	2.366	448	0.009	0.39 [0.16, 0.62]
	Apart	3.23 ± 0.77				
PI	Married	2.08 ± 0.54	2.209	448	0.014	0.39 [0.16, 0.62]
	Apart	1.89 ± 0.42				

Male students reported significantly higher parental involvement in family education (2.14 ± 0.56) than female students (1.98 ± 0.49), *t*(448) = 3.287, *p* < 0.001, Cohen’s d = 0.31, 95% CI [0.12, 0.50]. Males also showed advantages in both subjective wellbeing (males: 65.13 ± 25.41; females: 53.15 ± 24.61) *t*(448) = 5.081, *p* < 0.001, Cohen’s *d* = 0.48, 95% CI [0.29, 0.67], and psychological wellbeing (males: *M* = 3.62 ± 0.86; females: *M* = 3.41 ± 0.76), *t*(448) = 2.751, *p* = 0.003, Cohen’s *d* = 0.26, 95% CI [0.08, 0.44].

Parental marital status had a significant impact on parental educational involvement and children’s psychological status. Parents in married families demonstrated significantly higher educational involvement (2.08 ± 0.54) than those in divorced families (1.89 ± 0.42), *t*(448) = 2.209, *p* = 0.014, Cohen’s *d* = 0.39, 95% CI [0.16, 0.62]. Children from married families reported significantly better psychological wellbeing (*M* = 3.54 ± 0.82) than their peers from divorced families (*M* = 3.23 ± 0.77), *t*(448) = 2.366, *p* = 0.009, Cohen’s *d* = 0.39, 95% CI [0.16, 0.62]. In contrast, no significant difference was found in subjective wellbeing between the two groups (M-married = 59.70 ± 29.70; M-divorced = 54.70 ± 26.00), *t*(448) = 1.208, *p* = 0.114, Cohen’s *d* = 0.18, 95% CI [–0.04, 0.41].

#### 3.2.2 Effects of grade, birth order, co-residents, and parental education level

As shown in the analysis of variance (ANOVA) and *post-hoc* tests (see Table 2), the following results were obtained regarding the effects of the above variables.

**TABLE 2:** ANOVA of the effects of other demographic variables.

I.V. and D.V.	*Post-hoc* test group	Comparison group	*F*	*p*	Δ M	SD	η^2^*p*
Grade-PI			9.49	< 0.001			0.04
	Grade 7	Grade 8			0.17	0.05	
		Grade 9			0.27	0.06	
BO-PI			3.62	0.013			0.02
	Only child	First child			0.16	0.07	
		Second child			0.16	0.07	
		Third or above			0.44	0.16	
CR –SWB			4.05	0.007			0.03
	Parents	Mother only			11.35	3.48	
CR –PWB			3.08	0.027			0.02
	Parents	Mother only			0.31	0.11	
CR –PI			7.85	< 0.001			0.05
	Parents	Mother only			0.23	0.07	
		Father only			0.31	0.12	
		Other relatives			0.47	0.15	
FE -SWB			3.18	0.024			02
	High school or below	Bachelor’s/associate’s degree			–8.33	2.78	
FE-PWB			11.23	< 0.001			0.07
	Bachelor’s/associate’s degree	High school or below			0.47	0.09	
		Unaware			0.38	0.11	
	High school or below	Master or above			–0.40	0.15	
	Master or above	Unaware			0.52	0.17	
FE -PI			4.80	0.003			0.03
	High school or below	Bachelor’s/associate’s degree			–0.21	0.06	
ME -SWB			2.66	0.048			0.02
	Bachelor’s/associate’s degree	High school or below			6.13	2.86	
		Unaware			8.51	3.40	
ME -PWB			13.97	< 0.001			0.09
	Bachelor’s/associate’s degree	High school or below			0.49	0.00	
		Unaware			0.48	0.10	
	High school or below	Master or above			–0.53	0.16	
ME -PI			6.517	< 0.001			0.04
	High school or below	Bachelor’s/associate’s degree			–0.19	0.06	
		master or above			–0.39	0.10	
	master or above	unaware			0.29	0.11	

IV, Independent Variable; DV, Dependent Variable; BO, Birth Order; CR, Co-residents; FE, Father’s education level; ME, Mother’s education level.

There was a significant difference in parental involvement across different grades (*F* = 9.49, *p* < 0.001, η^2^*p* = 0.04). *Post-hoc* tests indicated that parental involvement among 7th-grade students was significantly higher than that among 8th-grade and 9th-grade students, while no significant difference was found between 8th-grade and 9th-grade students.

Birth order had a significant impact on parental involvement (*F* = 3.62, *p* = 0.013, η^2^*p* = 0.02). The parental involvement for only children was significantly higher than that for children with siblings. However, there was no significant difference in wellbeing across different birth orders.

Differences in co-residents exerted significant effects on subjective wellbeing (*F* = 4.05, *p* < 0.01, η^2^ *p* = 0.03), psychological wellbeing (*F* = 3.08, *p* < 0.05, η^2^*p* = 0.02), and parental involvement (*F* = 7.85, *p* < 0.001, η^2^*p* = 0.05). Students co-residing with both parents had significantly higher subjective wellbeing, psychological wellbeing, and parental involvement than those co-residing only with their mothers. Parental involvement for students co-residing with both parents was also significantly higher than that for students in other co-residence types.

Father’s education level significantly impacted students’ subjective wellbeing (*F* = 3.18, *p* < 0.05, η^2^*p* = 0.02), psychological wellbeing (*F* = 11.23, *p* < 0.001, η^2^*p* = 0.07), and parental involvement (*F* = 4.80, *p* < 0.01, η^2^*p* = 0.03). Students with fathers holding a bachelor’s or associate’s degree scored higher in all three aspects than those with fathers having high school education or below; students with fathers holding a master’s degree or above had higher psychological wellbeing than those with fathers having high school education or below, and those unaware of their fathers’ education level.

Mother’s education level also exerted significant effects on students’ subjective wellbeing (*F* = 2.66, *p* < 0.05, η^2^*p* = 0.02), psychological wellbeing (*F* = 13.97, *p* < 0.001, η^2^*p* = 0.09), and parental involvement (*F* = 6.51, *p* < 0.001, η^2^ = 0.04): students with mothers holding a bachelor’s/associate’s degree performed better in all three aspects than those with mothers having high school education or below; students with mothers holding a master’s degree or above had higher psychological wellbeing and parental involvement than those with mothers having high school education, and then those unaware of their mothers’ education level.

Means, standard deviations, and the correlation matrix of all variables are presented in Table 3. Correlation analysis results indicated that correlations existed both within and between the structures of the variables, and the magnitude and direction of the correlation coefficients were consistent with expectations.

**TABLE 3:** The correlation matrix (Spearman correlation).

Variables	1	2	3	4	5	6	7	8	9	10	11	12	13	14
1. Gender	1.00	0.07	0.004	–0.23**	0.24**	–0.05	–0.07	–0.10*	–0.10*	–0.12*	–0.10*	–0.10*	–0.12*	–0.13**
2. Grade		1	0.07	–0.06	–0.02	0.05	0.08	–0.004	–0.03	–0.01	–0.09	–0.17**	–0.21**	–0.19**
3. Parental married status			1.00	–0.06	–0.10*	–0.09	–0.09	–0.13**	–0.06	–0.09*	–0.07	–0.10*	–0.08	–0.10*
4. SWB				1.00	0.48**	0.48**	0.47**	0.47**	0.46**	0.52**	0.32**	0.37**	0.35**	0.41**
5. Autonomy					1.00	0.55**	0.60**	0.43**	0.50**	0.64**	0.27**	0.24**	0.21**	0.29**
6. Environmental mastery						1	0.59**	0.53**	0.55**	0.58**	0.29**	0.25**	0.26**	0.32**
7. Personal growth							1	0.58**	0.64**	0.69**	0.28**	0.25**	0.23**	0.29**
8. Positive relations with others								1	0.58**	0.61**	0.40**	0.35**	0.34**	0.43**
9. Purpose in life									1	0.61**	0.36**	0.33**	0.29**	0.39**
10. Self-acceptance										1	0.37**	0.29**	0.30**	0.38**
11. Attitudes toward PI											1	0.57**	0.53**	0.81**
12. Family activities												1	0.72**	0.87**
13. School activities													1	0.86**
14. Parental Involvement														1
M	1.49	1.78	1.09	59.22	3.18	3.66	3.81	3.49	3.41	3.55	2.32	1.89	1.97	2.06
SD	0.50	0.74	0.29	25.70	0.96	0.96	1.20	0.95	1.07	1.02	0.61	0.66	0.62	0.53

** Significant at 0.01 level (two-tailed);

* Significant at 0.05 level (two-tailed).

Specifically, gender was negatively correlated with subjective wellbeing, autonomy, agreeableness, purpose in life, self-acceptance, attitudes toward parental involvement, family activities, and school activities, which suggests that female students scored significantly lower than male students in these aspects. Grade was negatively correlated with family activities and school activities, implying that as students advanced to higher grades, the level of parental engagement with children in family activities and parental participation in school activities decreased. Parental marital status showed a significant negative correlation with students’ autonomy, agreeableness, self-acceptance, and family activities. Additionally, subjective wellbeing, the six dimensions of psychological wellbeing, and the three dimensions of parental involvement all exhibited significant positive correlations with one another.

### 3.3 Measurement model

#### 3.3.1 Validation of the measurement model for core latent variables

Before conducting the mediation effect test, confirmatory factor analysis (CFA) was used to test the measurement model. There were 3 latent variables employed in this study. Parental involvement, which included three indicators: attitude toward parental involvement, family activities, and school activities. psychological wellbeing consists of six indicators (autonomy, environmental mastery, personal growth, positive relations with others, purpose in life, and self-acceptance). subjective wellbeing comprised five items.

The measurement model yielded a borderline acceptable fit to the data, χ^2^(77) = 237.629, CFI = 0.89, TLI = 0.87, RMSEA = 0.08. Although the CFI and TLI values were slightly below the recommended cutoff of 0.90, the RMSEA fell within the marginally acceptable range (Alijev et al., 2021; Rogers et al., 2009). These results suggest that the model provides a reasonable approximation of the underlying construct, despite not meeting the more stringent fit criteria. This level of fit is common in educational research, particularly when scales are applied across different cultural or contextual settings (Wilson Fadiji and Reddy, 2023).

#### 3.3.2 Comparison of first-order vs. second-order factor models for parental involvement

To examine the multidimensional structure of parental involvement, two confirmatory factor models were compared:

First-order three-factor model: Attitude, family activities, and school activities were specified as three correlated latent variables. This model showed poor fit: χ^2^(368) = 1642.11, CFI = 0.82, TLI = 0.81, RMSEA = 0.07. The three latent variables were highly positively correlated (*r* ranged from 0.71 to 0.89, *p* < 0.001). This suggested the potential existence of a higher-order common factor.

Second-order single-factor model: Based on the first-order model, a second-order factor of “overall parental involvement” was added. It accounted for the common variance among the three dimensions. The fit of this model was significantly improved: χ^2^(367) = 1236.90, CFI = 0.89, TLI = 0.87, RMSEA = 0.07. A chi-square difference test confirmed that the second-order model had significantly better fit than the first-order model [Δχ^2^(1) = 405.21, *p* < 0.001]. As shown in Table 4, the standardized factor loadings of each indicator on its corresponding latent variable were significant (*p*s < 0.001). The correlation coefficients among the latent variables are presented in Table 5, revealing significant positive correlations between the three variables.

**TABLE 4:** Decomposition of the effects of overall parental involvement on subjective wellbeing.

Path	Effect type	β	95% Bootstrap CI
PI → SWB	Total effect	0.38	[0.26, 0.50]
PI → SWB	Direct effect	0.17	[0.05, 0.30]
PI → PWB → SWB	Indirect effect	0.21	[0.14, 0.29]

PI, overall parental involvement (second-order latent construct); PWB, psychological wellbeing; SWB, subjective wellbeing. All coefficients are standardized estimates. Bias-corrected bootstrap confidence intervals were based on 5,000 resamples. Effects are considered significant when the confidence interval does not include zero.

**TABLE 5:** Direct, indirect, and total effects of parental involvement dimensions on subjective wellbeing.

Dimension	Effect type	β	95% Bootstrap CI
Attitude toward PI	Total effect	0.283	[0.12, 0.44]
	Direct effect	–0.010	[–0.18, 0.18]
	Indirect effect	0.294	[0.18, 0.42]
Family activities	Total effect	0.207	[0.05, 0.41]
	Direct effect	0.148	[0.001, 0.31]
	Indirect effect	0.059	[–0.04, 0.16]
School activities	Total effect	0.019	[–0.13, 0.21]
	Direct effect	0.038	[–0.06, 0.18]
	Indirect effect	-0.019	[–0.10, 0.09]

Indirect effect paths are the dimensions → PWB → SWB. All path coefficients are standardized estimates. Effects are considered significant when the 95% bootstrap confidence interval does not include zero.

In the second-order model, the standardized factor loadings of the overall parental involvement factor on each dimension were all high and significant (Attitude toward parental involvement = 0.71, Family activities = 0.97, School activities = 0.81; all *p*s < 0.001). Residual correlations among the three factors were non-significant (| *r*| < 0.10). These results strongly supported the conceptualization of parental involvement as a higher-order global construct with three subdimensions. Therefore, this second-order factor was adopted in subsequent structural models testing the overall effect of parental involvement. In contrast, dimension mean scores were used as observed variables when exploring dimension-specific effects. Collinearity issues of these variables had been evaluated in the next section.

#### 3.3.3 Multicollinearity diagnosis for parental involvement dimensions as concurrent predictors

Since the subsequent analysis would incorporate the three dimensions of parental involvement as concurrent predictors into the model, multicollinearity was examined to ensure the stability of parameter estimates. The three dimensions exhibited moderate to high correlations (r ranged from 0.53 to 0.72). Further diagnosis revealed that the variance inflation factors (VIFs) of all three dimensions were at a low level (VIF = 1.387–2.271), far below the commonly used critical value of 5. The corresponding tolerance values ranged from 0.440 to 0.721, which were above the standard of 0.2. In addition, the maximum condition index was 12.24, lower than the threshold of 30. These indicators consistently demonstrated that multicollinearity would not substantially affect the stability of parameter estimates in this study.

### 3.4 Structural model

#### 3.4.1 Testing the mediating effect of psychological wellbeing

Given that both the overall effect parental involvement model and the dimension-specific parental involvement model demonstrated acceptable model fit and that multicollinearity diagnostics indicated no threat to the stability of parameter estimates, mediation analyses were conducted using both modeling approaches. This strategy allowed us to examine the overall mediating mechanism as well as the potentially distinct pathways associated with specific dimensions of parental involvement.

##### 3.4.1.1 Overall effect model

SEM was first employed to test the overall mediation model, in which parental involvement was specified as a second-order latent construct. The model demonstrated acceptable fit to the data, χ^2^(369) = 12,236.889, CFI = 0.88, TLI = 0.87, RMSEA = 0.07.

**FIGURE 2:**
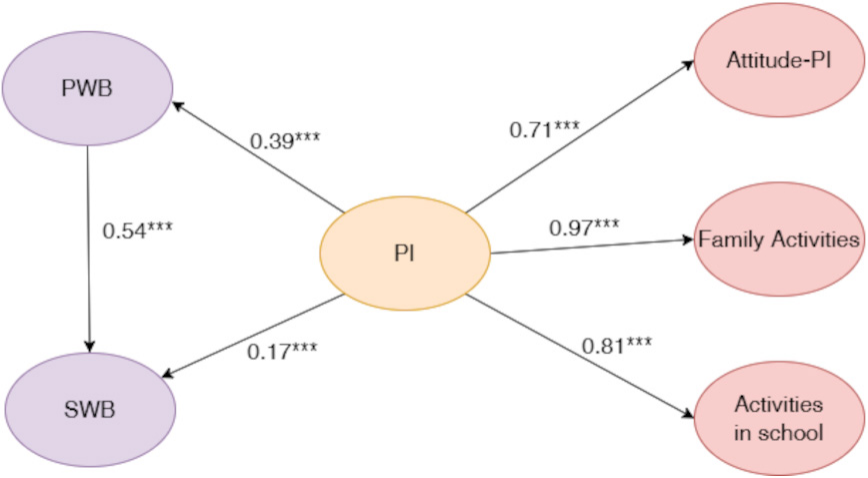
Standardized path coefficients for the mediated latent variable model. The figure illustrates the overall mediation model examining the associations among parental involvement (PI), psychological wellbeing (PWB), and subjective wellbeing (SWB). The model demonstrated acceptable fit to the data, χ^2^ (369) = 12,236.889, CFI = 0.88, TLI = 0.87, RMSEA = 0.07. All hypothesized structural paths were statistically significant. Overall parental involvement was positively associated with psychological wellbeing, which in turn was positively related to subjective wellbeing. The direct path from parental involvement to subjective wellbeing also remained significant, indicating a partial mediation pattern. Indirect effects were tested using bias-corrected bootstrap procedures with 5,000 resamples, confirming a significant indirect effect of parental involvement on subjective wellbeing via psychological wellbeing. Standardized path coefficients are presented in the figure. ***p < 0.001.

As illustrated in Figure 2, all hypothesized structural paths were statistically significant. Specifically, overall parental involvement positively predicted psychological wellbeing (PWB; β = 0.39, *p* < 0.001), which in turn was positively associated with subjective wellbeing (subjective wellbeing; β = 0.54, *p* < 0.001). In addition, the direct path from parental involvement to subjective wellbeing remained statistically significant (β = 0.17, *p* < 0.001).

To quantify the mediating role of psychological wellbeing, indirect effects were tested using bias-corrected bootstrap procedures with 5,000 resamples. As shown in Table 4, the indirect effect of overall parental involvement on subjective wellbeing via psychological wellbeing was statistically significant (β = 0.211), with a 95% bias-corrected confidence interval of [0.144, 0.287], which did not include zero. After accounting for the mediator, the direct effect of parental involvement on subjective wellbeing remained significant [β = 0.173, 95% CI [0.051, 0.295)]. The total effect of parental involvement on subjective wellbeing was 0.384 [95% CI (0.259, 0.501)]. Taken together, these results indicate a significant indirect effect alongside a remaining direct effect, suggesting that psychological wellbeing partially mediates the association between overall parental involvement and subjective wellbeing.

##### 3.4.1.2 Dimension-specific model

Although the overall model captures the general mechanism linking parental involvement, psychological wellbeing, and subjective wellbeing, this approach may obscure heterogeneity across different forms of parental involvement. To further examine whether specific dimensions of parental involvement contribute differentially to this mediating pathway, a dimension-specific SEM was estimated in which attitude toward parental involvement, family activities, and school activities were entered simultaneously as correlated but distinct predictors.

**FIGURE 3:**
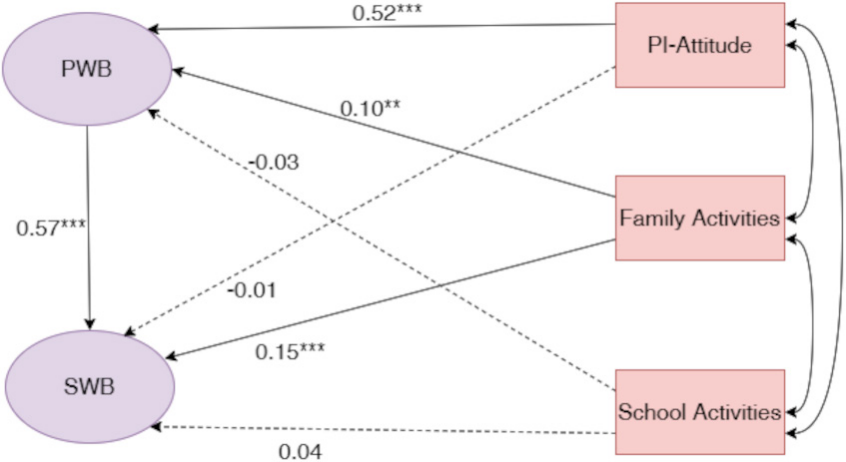
Standardized regression coefficients (β) for the dimension-specific path model. The model examined attitudes toward parental involvement, activities at home, and activities at school as correlated predictors of psychological wellbeing (PWB) and subjective wellbeing (SWB). Model fit was acceptable for exploratory purposes, χ^2^ (368) = 1,642.11, CFI = 0.82, TLI = 0.81, RMSEA = 0.088. Consistent with the overall model, PWB was a strong predictor of SWB. Attitudes toward parental involvement showed the strongest association with PWB, activities at home exhibited smaller but significant effects, whereas activities at school were not significantly related to PWB. Activities at home also demonstrated a direct association with SWB, indicating dimension-specific pathways linking parental involvement to adolescent wellbeing. **p < 0.01; ***p < 0.001.

The dimension-specific model showed weaker, yet acceptable for exploratory purposes, fit to the data, χ^2^(368) = 1,642.11, CFI = 0.82, TLI = 0.81, RMSEA = 0.088. Given the exploratory aim of disentangling dimension-level pathways, the model was retained to examine differential patterns across parental involvement components (see Figure 3).

Consistent with the overall model, psychological wellbeing remained a strong predictor of subjective wellbeing (β = 0.57, *p* < 0.001). However, the three parental involvement dimensions displayed distinct patterns of association with both psychological wellbeing and subjective wellbeing.

With respect to psychological wellbeing, students’ attitudes toward parental involvement emerged as the strongest predictor (β = 0.52, *p* < 0.001), indicating that students’ perception of parental involvement is closely linked to their psychological functioning. Family activities also positively predicted psychological wellbeing, albeit with a smaller effect size (β = 0.10, *p* < 0.05). When the three dimensions of parental involvement were entered simultaneously into the SEM, school-based involvement did not show a statistically significant unique association with student wellbeing (β = -0.03, *p* = 0.48), after accounting for the shared variance with family activity involvement and parental attitudes. This finding suggested that its association with adolescents’ psychological wellbeing may largely reflect variance shared with other forms of parental involvement rather than a distinct dimension-specific effect.

Regarding subjective wellbeing, family activities showed a significant direct association with subjective wellbeing (β = 0.15, *p* < 0.001), indicating that shared family activities may contribute to adolescents’ affective wellbeing beyond their association with psychological wellbeing. The direct paths from attitudes toward parental involvement (β = -0.01) and school activities (β = 0.04) to subjective wellbeing were not statistically significant (all *p*s > 0.10).

Bias-corrected bootstrap analyses (5,000 resamples) were conducted to estimate the direct, indirect, and total effects of each parental involvement dimension on subjective wellbeing (see Table 5). The results revealed qualitatively different mediating patterns across dimensions. Regarding students’ attitudes toward parental involvement, the total effect on subjective wellbeing was statistically significant. This effect was fully mediated by psychological wellbeing [β = 0.294, 95% CI [0.179, 0.420)], as the direct effect was not significant. In contrast, the total effect of family activity involvement on subjective wellbeing was primarily attributable to its direct association with subjective wellbeing, while the indirect effect via psychological wellbeing was not statistically significant. Finally, school activity involvement showed no significant total, direct, or indirect effects on subjective wellbeing within the simultaneous dimension-specific model.

#### 3.4.2 The moderating role of gender in the mediation process

To examine whether the mediating role of psychological wellbeing varied by gender, moderation and conditional process analyses were conducted. Given the complexity of the proposed model, moderation was primarily tested using interaction-based conditional process modeling, supplemented by multi-group SEM analyses to examine gender differences in structural paths.

##### 3.4.2.1 Gender differences in the overall mediation model

Multi-group structural equation modeling (MG-SEM) was first conducted to explore whether the structural paths in the overall mediation model differed between male (*n* = 228) and female students (*n* = 222). Table 6 showed the results of measurement and structural invariance testing across gender groups. Although the chi-square difference test indicated a significant decrease in model fit when measurement weights were constrained (Δχ^2^ = 75.91, Δdf = 24, *p* < 0.001), changes in approximate fit indices were relatively small (ΔCFI = 0.01; ΔRMSEA = 0.00). Further constraining structural paths also led to a significant decrease in model fit (Δχ^2^ = 207.48, Δdf = 58, *p* < 0.001), changes in approximate fit indices were relatively small (ΔCFI = 0.01; ΔRMSEA = 0.00). As the sensitivity of the chi-square statistic to sample size, measurement and structural invariance were evaluated using a combination of chi-square difference tests and changes in approximate fit indices. Following established recommendations (Chen, 2007), changes in CFI (ΔCFI ≤ 0.01) and RMSEA (ΔRMSEA ≤ 0.015) were used as primary criteria. Although chi-square differences were statistically significant, changes in CFI and RMSEA were minimal. This indicated partial but acceptable measurement and structural non-invariance across gender.

**TABLE 6:** Measurement and structural invariance testing across gender groups.

Model	χ ^2^	df	χ ^2^/df	CFI	TLI	RMSEA	Δ χ ^2^(Δ df)	Δ CFI	Δ RMSEA
Mboys	2134.6	738	2.89	0.87	0.86	0.05	–	–	–
Mgirls	2275.314	738	3.08	0.86	0.85	0.06	–	–	–
M1 unconstrained	1935.08	738	2.62	0.84	0.82	0.06	–	–	–
M2 measurement weights	2010.99	762	2.64	0.83	0.82	0.06	75.91(24)	0.01	0.00
M3 structural weights	2142.56	796	2.69	0.82	0.82	0.06	131.57 (34)	0.01	0.00

M1 = unconstrained multi-group model; M2 = measurement weights constrained equal; M3 = structural weights further constrained equal.Δχ^2^ (Δdf),ΔCFIΔCFI, and ΔRMSEA indicate changes from the previous model and are used to test measurement and structural invariance.***p < 0.001.

The results of MG-SEM path estimates across gender were shown in Table 7A. Inspection of individual paths revealed that the direct association between parental involvement and psychological wellbeing differed by gender (CR = 2.73, *p* < 0.01). This direct effect was statistically significant among female students (β = 0.16, *p* < 0.05) but not among male students (β = 0.09, *p* > 0.05). In contrast, the path from parental involvement to subjective wellbeing (CR = 1.26) and the path from psychological wellbeing to subjective wellbeing (CR = 1.90) did not significantly differ between genders.

**TABLE 7A:** Multi-group SEM path estimates across gender.

Path	Male β	Female β	CR	Group difference
PI → PWB	0.25***	0.51***	2.73	Not significant
PI → SWB	0.09	0.16*	1.26	Significant
PWB → SWB	0.47***	0.63***	1.90	Not significant

All coefficients are standardized estimates. CR values greater than | 1.96| indicate significant between-group differences.

To further examine whether the indirect effect of parental involvement on subjective wellbeing via psychological wellbeing differed by gender, conditional indirect effects were estimated using PROCESS Model 7 with 5,000 bootstrap resamples (see Table 7B). The indirect effect was statistically significant for both male [*B* = 0.21, 95% CI (0.11, 0.34)] and female students [*B* = 0.28, 95% CI (0.19, 0.39)].Importantly, the index of moderated mediation was statistically significant [Index = 4.27, 95% CI (0.19, 8.61)], indicating that gender significantly moderated the indirect effect in the overall model.

**TABLE 7B:** Conditional indirect effects of overall parental involvement by gender.

Gender	Indirect effect (B)	Boot SE	95% Bootstrap CI
Male	0.21	0.06	[0.11, 0.34]
Female	0.28	0.07	[0.19, 0.39]
Index of moderated mediation	Index	Boot SE	95% Bootstrap CI
Gender	4.27	2.16	[0.19, 8.61]

Conditional indirect effects were estimated using PROCESS Model 7 with 5,000 bootstrap resamples. Coefficients are unstandardized. A moderated mediation effect is inferred when the confidence interval for the index does not include zero.

These results collectively demonstrate that gender moderates the indirect pathway from parental involvement to subjective wellbeing through psychological wellbeing, with a stronger effect for female students. This moderated mediation is primarily attributable to the stronger direct effect of parental involvement on psychological wellbeing among females, as shown by both multi-group path comparisons and the significant index of moderated mediation.

##### 3.4.2.2 Gender as a moderator in the dimension-specific models

Given evidence that different dimensions of parental involvement operate through distinct mechanisms, conditional process analyses were further conducted at the dimension level using PROCESS Model 7 (Hayes, 2017), with gender specified as a moderator of the first-stage path. Bias-corrected bootstrap confidence intervals were estimated using 5,000 resamples (see Table 8).

**TABLE 8:** Conditional indirect effects of parental involvement dimensions by gender.

Dimensions	Gender	Indirect effect (B)	Boot SE	Bootstrap 95% CI
Attitude-PI	Male	8.16	1.99	[4.72, 12.47]
	Female	9.98	1.81	[6.44, 13.53]
	Index (Gender)	1.81	2.72	[–3.94, 6.86]
Family activities	Male	2.73	1.23	[0.55, 5.45]
	Female	12.03	1.85	[8.55, 15.75]
	Index (Gender)	9.31	2.21	[4.99, 13.71]
School activities	Male	2.94	1.32	[0.59, 5.73]
	Female	10.37	1.87	[6.88, 14.19]
	Index (Gender)	7.43	2.30	[3.02, 11.94]

Conditional indirect effects were estimated using PROCESS Model 7 with 5,000 bootstrap resamples. Coefficients are unstandardized. Gender was coded as 0 = male, 1 = female.

For students’ attitude toward parental involvement, the index of moderated mediation was not statistically significant [Index = 1.81, 95% CI (-3.94, 6.86)], indicating that the indirect effect via psychological wellbeing did not differ by gender. The indirect effects were statistically significant and of comparable magnitude for both male and female students, whereas the direct effect was significant only among female students.

In contrast, gender significantly moderated the indirect effects associated with behavioral forms of parental involvement. For family activity involvement, the index of moderated mediation was statistically significant [Index = 9.31, 95% CI (4.99, 13.71)], indicating stronger indirect effects for female students. Similarly, for school activity involvement, the index of moderated mediation was significant [Index = 7.43, 95% CI (3.02, 11.94)], again suggesting that the indirect association via psychological wellbeing was more pronounced among female students.

Overall, these findings indicate that gender differences in the mediation process are dimension-specific rather than universal. Gender appears to play a moderating role primarily in behavioral forms of parental involvement (i.e., family and school activities), whereas the attitudinal dimension operates through a largely similar psychological mechanism for male and female students.

## 4 Discussion

### 4.1 Current level of wellbeing and its influencing factors

Adolescents in this study reported a moderate level of overall wellbeing, with notable individual differences, indicating the influence of multiple demographic and familial factors.

Consistent with prior research (Newland et al., 2019), male students reported higher wellbeing than female students. Greater emotional sensitivity and stress reactivity during adolescence may render females more vulnerable to academic and family-related pressures, thereby lowering wellbeing levels (Garcia et al., 2017).

Family structure also played an important role. Adolescents living with both parents reported the highest wellbeing, supporting ecological systems theory, which emphasizes the family as a central developmental context (Tudge et al., 2016). Dual-parent co-residence may provide more stable emotional support and coordinated parenting, with paternal presence showing particular benefits, consistent with earlier findings (Chung et al., 2020; Clayborne et al., 2023).

Parental marital status was unrelated to subjective wellbeing but was negatively associated with psychological wellbeing, especially in autonomy, positive relations, and self-acceptance. This suggests that psychological wellbeing may be more sensitive to long-term family structure, whereas subjective wellbeing reflects more immediate emotional experiences (Garcia et al., 2017; Hayes, 2017).

Higher parental education was also linked to greater adolescent wellbeing, likely reflecting advantages in socioeconomic resources and parenting practices that foster supportive developmental environments (Chung et al., 2020; Clayborne et al., 2023).

These findings underscore a key distinction between subjective and psychological wellbeing: The former appears more responsive to short-term emotional states, whereas the latter is more closely tied to stable family contexts and parenting conditions.

### 4.2 The influencing factors of parental involvement

In this study, parental involvement was significantly influenced by factors including gender, grade, birth order, parental marital status, cohabitants, and parental education level. Specific findings are as follows:

Gender significantly influences parental involvement. The scores of parental involvement were for male students than for female students. This finding is consistent with existing research (Careemdeen, 2024; Wilson Fadiji and Reddy, 2023). It may be attributed to parents’ traditional gender role perceptions, as they tend to invest more educational resources in boys to meet their higher academic expectations. Meanwhile, such perceptions also assume differences in learning initiative between boys and girls. In the minds of Chinese parents, most boys are perceived as having lower initiative and requiring more parental supervision to meet school requirements. In contrast, girls are generally more obedient, more likely to follow teachers’ instructions, and have higher initiative, making parents more assured.

Grade significantly influences parental involvement. The scores of parental involvement were higher for 7th-grade students than for students in the other two grades. This aligns with existing research (Liu, 2023), reflecting the “critical transition period” phenomenon of parental involvement. As adolescents’ self-awareness awakens, they deliberately resist excessive parental involvement in their academic lives after entering junior high school (Hill et al., 2018; Salinas et al., 2019). However, junior high school curricula involve more subjects and increased difficulty, which also poses a challenge for parents. Faced with such resistance and challenges, parents must adopt different approaches to educational involvement than in primary school to adapt to students’ psychological development and academic requirements after they enter junior high.

Birth order significantly influences parental involvement. Parents in one-child families showed significantly higher levels of involvement compared to those in other birth orders. This is consistent with existing research (Zhu et al., 2023). In one-child families, all family resources can be concentrated on a single child, and family capital can be better utilized to form an aggregation effect. With each additional child, family resources are naturally dispersed, leading to a decrease in parental involvement.

Co-resident significantly influences parental involvement. Compared to those living with only one parent, students living with both parents had significantly higher parental involvement scores. This result is consistent with family systems theory (Brown et al., 2021). In a stable family structure, each member can fulfill their unique role. Complementary and cooperative functions between parents are more fully realized. Through role division, parents can provide more involvement opportunities and stronger emotional support.

Parents’ educational attainment significantly influences parental involvement. Involvement was significantly higher when parents held an associate’s degree, bachelor’s degree, or above, compared to parents with a high school education or less. This may be because highly educated parents have benefited more from the education system themselves. They can better understand how the system operates and place a higher value on education. Furthermore, they tend to have stronger educational self-efficacy. They believe their involvement can positively impact their children’s learning, making them more willing to actively participate in school-related activities (Yin et al., 2025).

### 4.3 Mediation effect of psychological wellbeing between parental involvement and subjective wellbeing

By integrating both the overall and dimension-specific models, the present study provides a nuanced account of the pathways through which parental involvement is associated with adolescents’ subjective wellbeing. At the overall level, parental involvement was linked to subjective wellbeing through both a direct pathway and an indirect pathway via psychological wellbeing, indicating a pattern consistent with partial mediation. Notably, the indirect pathway through psychological wellbeing accounted for a substantial proportion of the total association, suggesting that parental involvement may be related to adolescents’ wellbeing not only through immediate affective experiences but also through broader psychological functioning.

This pattern is broadly consistent with self-determination theory, which posits that social contexts supportive of autonomy, competence, and relatedness are conducive to both hedonic wellbeing and eudaimonic functioning (Ryan and Deci, 2001). Rather than indicating causality, these findings suggest that parental involvement may be linked to adolescent subjective wellbeing partly through its connection to psychological wellbeing. This helps bridge previous research that has often focused separately on either short-term emotional outcomes or longer-term developmental indicators.

Importantly, the dimension-specific analyses revealed marked heterogeneity in these pathways. Within the framework of family–school systems theory (Ryan and Adams, 1995), students’ attitude toward parental involvement emerged as a particularly salient explanatory pathway. Its association with subjective wellbeing was fully accounted for by psychological wellbeing in the present model, with no additional direct association observed. This pattern aligns with prior work suggesting that adolescents’ subjective interpretations of parental involvement may be more consequential for their wellbeing than the objective frequency of parental behaviors (Thomas et al., 2020). From a self-determination perspective, parental involvement may be most strongly linked to adolescents’ wellbeing when it is perceived as autonomy-supportive and is psychologically internalized by adolescents themselves.

In contrast, involvement through family activities exhibited a distinct pattern, characterized primarily by a direct correlation with subjective wellbeing. This finding suggests that shared family activities may be linked to adolescents’ wellbeing through more immediate affective experiences, such as enjoyment or emotional closeness. These experiences may operate relatively independently of broader evaluations of psychological wellbeing. Consistent with family systems perspectives (Rothbaum et al., 2002), the family has long been viewed as a primary context for emotional support and day-to-day interaction, providing opportunities for affective engagement that can directly shape adolescents’ subjective experience.

It is important to note that the non-significant path from school-based involvement to student wellbeing in the structural model should not be interpreted as indicating a lack of association per se. As shown in the bivariate correlations (Table 3), school-based involvement is positively associated with student wellbeing. However, in the dimension-specific model, all three highly correlated dimensions were entered simultaneously, such that each path represents the unique effect of a given dimension after controlling for the others. Under this specification, the shared variance among behavioral and attitudinal forms of parental involvement is partialled out, which may attenuate the unique contribution of school-based involvement. This pattern suggests that school-based involvement may influence student wellbeing primarily through mechanisms shared with other forms of parental involvement, rather than through a distinct pathway. As family-school systems theories emphasize (Ryan and Adams, 1995), the potential benefits of school-based involvement depend on family-school coordination and mutual understanding.

Meanwhile, it is also important to note that these findings were obtained within the Chinese educational and family context. In China, parental involvement is often shaped by strong academic expectations and normative family obligations. Such cultural characteristics may influence how adolescents perceive and internalize different forms of parental involvement, and should be considered when interpreting the results.

### 4.4 The moderating role of gender in the mediating model

By integrating multi-group structural equation modeling with conditional indirect effect analyses, the present study examined whether gender was associated with variability in the pathways linking parental involvement, psychological wellbeing, and subjective wellbeing. Both analytic approaches converge to show that gender differences in these associations are selective. Their presence depends on the specific level and form of parental involvement examined. Therefore, gender appears to function as a contextual factor that shapes the strength of specific pathways within the model, rather than altering the overall structure of the relationships, which remained invariant across gender despite differences in effect magnitude.

At the overall level, parental involvement was more strongly associated with subjective wellbeing among female adolescents than among male adolescents. This difference mainly appeared in the indirect pathway through psychological wellbeing, as reflected by a statistically significant index of moderated mediation. The links from parental involvement to psychological wellbeing, and from psychological wellbeing to subjective wellbeing, showed no major gender differences at the level of individual path coefficients. This pattern does not mean the underlying psychological processes are different. Instead, it suggests that for female adolescents, psychological wellbeing may be a key explanatory pathway. It amplifies the overall connection between parental involvement and their subjective wellbeing.

The dimension-specific analyses further clarified the boundaries of this gender-related variability. Gender differences were evident in behavior-oriented forms of involvement (i.e., family activities and school). However, no such differences emerged in adolescents’ attitudinal evaluations of parental involvement. Specifically, the indirect link between behavioral involvement and subjective wellbeing, mediated by psychological wellbeing, was stronger among female adolescents. In contrast, the students’ attitude toward parental involvement showed no comparable gender difference. This pattern suggests that observable parental behaviors may be more susceptible to gendered interpretations than adolescents’ overall evaluations of parental involvement.

These findings are broadly consistent with social role theory. The theory emphasizes that gender differences in interpersonal sensitivity, emotional orientation, and relational expectations emerge through socialization processes rather than reflecting inherent psychological distinctions (Wood and Eagly, 2012). Prior research has shown that female adolescents tend to place greater emphasis on relational cues and emotional connectedness in close relationships, including family interactions (Wilson Fadiji and Reddy, 2023). From this perspective, observable parental behaviors (e.g., shared family activities or school engagement) may be more readily interpreted by female adolescents as expressions of care, emotional availability, and relational investment. This, in turn, may align these behaviors more closely with psychological wellbeing indicators such as relatedness and self-worth (Putra et al., 2023). In contrast, male adolescents may interpret similar behaviors through alternative frames, such as guidance, monitoring, or instrumental support. This difference in perspective may weaken the link between those behaviors and psychological wellbeing.

By contrast, adolescents’ attitudes toward parental involvement are shaped through the accumulation of repeated interactions over time and capture adolescents’ generalized sense of whether parental involvement is legitimate, caring, and supportive. Because this evaluative process operates at a more abstract cognitive level, it is less directly tied to the interpretation of particular parental actions and therefore less susceptible to momentary or behavior-specific gendered expectations. According to self-determination theory, these attitudes primarily signal adolescents’ perceived acceptance, belongingness, and autonomy support within the family context needs which are widely regarded as fundamental and relatively invariant across gender. Consistent with prior research indicating that autonomy-supportive parental orientations tend to be associated with adolescents’ psychological adjustment in a largely gender-invariant manner (Careemdeen, 2024), the present findings suggest that once parental involvement is cognitively integrated into a stable attitudinal framework, its association with psychological wellbeing may operate similarly for male and female adolescents.

These findings indicate that gender differences in the links between parental involvement and adolescent wellbeing are dimension-specific rather than pervasive. Gender appears to be more relevant for how adolescents interpret and internalize concrete parental behaviors than for how they evaluate parental involvement at a more abstract attitudinal level. It should be noted, however, that the present findings are based on cross-sectional data and therefore cannot establish temporal ordering or causal direction.

### 4.5 Educational and practical implications

The present findings have several implications for educational and family practice. Most notably, they highlight the importance of the quality of parental involvement rather than its sheer frequency. Parental involvement appears to be more strongly associated with adolescents’ wellbeing when it is perceived as supportive, respectful, and autonomy-enhancing, and when adolescents are able to internalize such involvement as a meaningful form of care. Accordingly, efforts to promote adolescent wellbeing may benefit from shifting attention from how much parents are involved to how their involvement is experienced and interpreted by adolescents.

From a practical perspective, parents and educators may consider emphasizing involvement practices that foster adolescents’ sense of acceptance, autonomy, and relatedness. Shared activities that allow for emotional connection and mutual engagement may contribute directly to adolescents’ positive experiences. Supportive parental attitudes may facilitate broader psychological wellbeing by shaping how adolescents make sense of parental involvement. Schools may play a facilitative role by encouraging family–school interactions that prioritize dialogue and adolescents’ subjective experiences, rather than focusing solely on behavioral compliance or academic monitoring. Importantly, these implications should be interpreted cautiously, given the correlational nature of the data, and future intervention research is needed to examine how autonomy-supportive involvement practices can be effectively cultivated in diverse family contexts.

## 5 Limitations and future study

Several limitations of the present study should be acknowledged. First, the cross-sectional design limits inferences regarding temporal ordering and causality among parental involvement, psychological wellbeing, and subjective wellbeing. Although the proposed model is theoretically informed, longitudinal or experimental research is needed to clarify the direction and potential reciprocity of these associations.

Second, the sample was drawn from a specific cultural and educational context in China, which may limit the generalizability of the findings to other sociocultural settings. Parental involvement in China is often embedded in strong academic norms and family expectations, which may shape adolescents’ perceptions differently than in other contexts. Meanwhile, although most subscales demonstrated acceptable internal consistency, the reliability of certain dimensions of psychological wellbeing was relatively modest (Cronbach’s α≈0.62). This limitation may have attenuated the estimated associations involving psychological wellbeing, suggesting that the observed effects could represent conservative estimates. Previous studies using Chinese samples have similarly reported lower reliability for this dimension, possibly reflecting cultural differences in the interpretation of self-evaluative items. Future research should examine whether the observed mechanisms replicate across cultural and educational systems.

Third, conducting both exploratory factor analysis (EFA) and confirmatory factor analysis (CFA) on the same sample may introduce overfitting and capitalize on chance characteristics of the data. Although we supplemented these analyses with procedural controls during data collection, future research should validate the measurement model using independent samples for EFA and CFA to enhance robustness. Finally, although gender differences were observed in specific pathways, the psychological mechanisms underlying these differences were not directly examined. Future research may explore factors such as gender role identification, parent–child communication patterns, or cultural expectations to further explain how gender shapes adolescents’ interpretations of parental involvement.

This study advances understanding of parental involvement by differentiating its attitudinal and behavioral components and highlighting the role of psychological wellbeing as an explanatory pathway. Future research may extend this work by examining how parental involvement is internalized over time and by identifying contextual factors that shape adolescents’ interpretive processes across diverse family and educational settings.

## 6 Conclusion

This study examined the associations between parental involvement and middle school students’ subjective wellbeing, focusing on the mediating role of psychological wellbeing and the moderating role of gender. The findings indicate that parental involvement is systematically associated with adolescents’ wellbeing, with psychological wellbeing serving as a key explanatory pathway. Gender was related to variability in the strength of specific pathways, particularly for behavior-oriented forms of involvement, rather than to differences in the overall model structure. By differentiating between attitudinal and behavioral dimensions of parental involvement, this study highlights the importance of adolescents’ perceived parental involvement in shaping wellbeing outcomes. Although the findings are correlational, they contribute to a more nuanced understanding of how parental involvement relates to adolescent wellbeing and offer empirically informed directions for future research and educational practice.

## Data Availability

The raw data supporting the conclusions of this article will be made available by the authors, without undue reservation.
